# Adenosine Signaling Mediates Osteogenic Differentiation of Human Embryonic Stem Cells on Mineralized Matrices

**DOI:** 10.3389/fbioe.2015.00185

**Published:** 2015-11-10

**Authors:** Vikram Rao, Yu-Ru V. Shih, Heemin Kang, Harsha Kabra, Shyni Varghese

**Affiliations:** ^1^Department of Bioengineering, University of California San Diego, La Jolla, CA, USA; ^2^Materials Science and Engineering Program, University of California San Diego, La Jolla, CA, USA

**Keywords:** human embryonic stem cells, osteogenic differentiation, mineralized matrix, calcium phosphate, A2b adenosine receptor

## Abstract

Human embryonic stem cells (hESCs) are attractive cell sources for tissue engineering and regenerative medicine due to their self-renewal and differentiation ability. Design of biomaterials with an intrinsic ability that promotes hESC differentiation to the targeted cell type boasts significant advantages for tissue regeneration. We have previously developed biomineralized calcium phosphate (CaP) matrices that inherently direct osteogenic differentiation of hESCs without the need of osteogenic-inducing chemicals or growth factors. Here, we show that CaP matrix-driven osteogenic differentiation of hESCs occurs through A2b adenosine receptor (A2bR). The inhibition of the receptor with an A2bR-specific antagonist attenuated mineralized matrix-mediated osteogenic differentiation of hESCs. In addition, when cultured on matrices in an environment deficient of CaP minerals, exogenous adenosine promoted osteogenic differentiation of hESCs, but was attenuated by the inhibition of A2bR. Such synthetic matrices that intrinsically support osteogenic commitment of hESCs are not only beneficial for bone tissue engineering but can also be used as a platform to study the effect of the physical and chemical cues to the extracellular milieu on stem cell commitment. Insights into the cell signaling during matrix-induced differentiation of stem cells will also help define the key processes and enable discovery of new targets that promote differentiation of pluripotent stem cells for bone tissue engineering.

## Introduction

Human embryonic stem cells (hESCs) have tremendous potential as a cell source for regenerative medicine due to their self-renewal and differentiation ability (Wobus and Boheler, 2005). One of the major areas of regenerative medicine includes the application of stem cells in tissue engineering and reconstruction (Heng et al., 2004; Marolt et al., 2012). However, a main challenge in using pluripotent stem cells (PSCs) lies in consistently directing them toward a targeted phenotype (Murry and Keller, 2008; De Peppo et al., 2013). This often requires complex stepwise *in vitro* strategies to direct differentiation of PSCs (Levi et al., 2012; Li and Niyibizi, 2012; De Peppo et al., 2013; Hynes et al., 2014; Phillips et al., 2014).

Biomaterials containing calcium phosphate (CaP) moieties have been recognized for their osteoinductive and osteoconductive functions and hence been widely used as a scaffold for bone tissue engineering both *in vitro* and *in vivo* (Yuan et al., 2007; Levi et al., 2012; Eyckmans et al., 2013; Inzana et al., 2014; Kobayashi et al., 2014). Recently, we have engineered biomineralized CaP matrices that intrinsically induce osteogenic differentiation of human mesenchymal stem cells (hMSCs), hESCs, and human-induced pluripotent stem cells (hiPSCs), without the use of osteoinductive soluble factors, such as BMPs or dexamethasone (Phadke et al., 2012; Kang et al., 2014a,b). These biomineralized matrices also support *in vivo* bone tissue formation, even in the absence of any exogenous biologics (Phadke et al., 2013; Kang et al., 2014b; Shih et al., 2015; Wen et al., 2015).

Calcium phosphate-based biomaterials promote osteogenic differentiation of progenitor or stem cells through multiple mechanisms. This involves the ability of CaP minerals to sequester osteoinductive growth factors, such as bone morphogenetic proteins, and/or regulate extracellular Ca^2+^ and ${\text{PO}}_{4}^{3-}$ concentrations (Autefage et al., 2009; Lee et al., 2011). This is further supported by the findings of significantly increased osteogenic differentiation of stem cells when cultured in medium containing high levels of Ca^2+^ and ${\text{PO}}_{4}^{3-}$ (Chai et al., 2011; Phadke et al., 2012). Moreover, it has been shown that biomaterials of CaP that can easily dissociate into Ca^2+^ and ${\text{PO}}_{4}^{3-}$ can contribute to better bone healing (Yuan et al., 2001; Barradas et al., 2013). In addition, a study by Wen et al. (2012) has demonstrated the involvement of L-type Ca^2+^ channels on Ca^2+^-mediated osteogenic differentiation. Recently, we have shown that the ${\text{PO}}_{4}^{3-}$ of the CaP minerals can promote osteogenic differentiation through A2b adenosine receptor (A2bR) signaling (Shih et al., 2014). This finding is consistent with other studies that demonstrated the role of adenosine signaling on bone tissue formation and osteogenesis of progenitor cells (Costa et al., 2011; Takedachi et al., 2012). For instance, studies by Evans et al. (2006) have shown the involvement of P1 purinergic adenosine receptor signaling in bone function. Specifically, it has been demonstrated that A2bR is functionally present in osteoprogenitor cells and plays a role in osteoblastic differentiation (Gharibi et al., 2011). Similarly, studies by Carroll et al. (2012) have shown that A2bR knockout mice had MSCs with decreased osteogenic potential, lower bone density, and delayed fracture repair (Carroll et al., 2012). Although it has been shown that osteogenesis of hMSCs is mediated by the activation of A2bR, its role in promoting osteogenic differentiation of PSCs, such as hESCs, remains unclear.

In this study, we determine whether the mineralized matrix-induced osteogenic differentiation of hESCs involves adenosine signaling similar to hMSCs. HESCs exhibit a developmentally naive phenotype as well as possess a vastly different cell machinery compared to hMSCs (Ulloa-Montoya et al., 2007; Aranda et al., 2009; Barbet et al., 2011). Despite the intrinsic differences between both cell types, we find that A2bR is involved in upregulation of genes associated with osteogenesis and increased protein expression of osteocalcin (OCN). This underscores the importance of A2bR signaling during osteogenic differentiation of stem cells with different maturation states.

## Materials and Methods

### PEGDA*-co*-A6ACA Hydrogel Synthesis

Poly(ethylene glycol)-diacrylate (PEGDA; *M*_n_ = 6 kDa), *N*-acryloyl 6-aminocaproic acid (A6ACA), and PEGDA-*co*-A6ACA hydrogels were synthesized, as described previously (Kang et al., 2014b). To summarize, 555 mg of A6ACA was dissolved in 3 mL of 1M NaOH. Upon neutralization, 60 mg of PEGDA was added to yield a precursor solution composed of 1M A6ACA and 2% (wt/vol) PEGDA. Using 0.15% N, N, N′, N′-tetramethylethylenediamine (TEMED) and 0.5% ammonium persulfate (APS) as the initiator and accelerator, respectively, the resulting solution was polymerized within 1-mm glass spacer plates (Bio-Rad, catalog number: 165-3311) for 1 h at room temperature (RT) to yield PEDGA-*co*-A6ACA hydrogels. The 1-mm hydrogel sheets were immersed and equilibrated in phosphate buffered saline (PBS; pH = 7.4) for 30 min, after which circular disks measuring 1 cm^2^ in area were punched out and incubated overnight in PBS.

### Hydrogel Mineralization and Sterilization

Mineralization of PEGDA-*co*-A6ACA hydrogels was carried out as described elsewhere (Phadke et al., 2012). Briefly, hydrogels were equilibrated in deionized (DI) water for 6 h and subsequently immersed in modified simulated body fluid (m-SBF; pH = 7.4) for 6 h. The ionic concentrations of m-SBF include 142.0 mM Na^+^, 5.0 mM K^+^, 2.5 mM Ca^2+^, 1.5 mM Mg^2+^, 103.0 mM Cl^−^, 10.0 mM ${\text{HCO}}_{3}^{-}$, 1.0 mM ${\text{HPO}}_{4}^{2-}$, and 0.5 mM ${\text{SO}}_{4}^{2-}$ (Oyane et al., 2003). After briefly rinsing in DI water, the hydrogels were immersed in a solution (pH = 5.2) containing 40 mM Ca^2+^ and 24 mM ${\text{HPO}}_{4}^{2-}$ while rotating on a VWR Mini-shaker at a speed of 200 rpm for 45 min at 25°C. Afterwards, the hydrogels were briefly rinsed in DI water and reimmersed in m-SBF for 48 h at 37°C, during which the solution was changed daily. The hydrogels were then immersed and equilibrated in PBS for 6 h prior to sterilization. Sterilization for both mineralized and non-mineralized PEGDA-*co*-A6ACA hydrogels was carried out by immersion in 70% ethanol for 3 h, followed by five daily washes in sterile PBS for 3 days prior to cell culture.

### Scanning Electron Microscopy and Energy Dispersive Spectra

Scanning electron microscopy (SEM) was performed on flat strips of mineralized and non-mineralized matrices. The samples were flash-frozen in liquid nitrogen, lyophilized overnight, and coated with Iridium for 7 s within a sputter (Emitech, K575X). The samples were imaged using scanning electron microscope (Philips XL30 ESEM), and also analyzed for elemental spectra using its integrated energy dispersive spectra (EDS) system. The Ca/P atomic ratio was computed using Oxford Energy Dispersive Spectra with INCA software.

### Cell Culture

Human embryonic stem cells (HUES9) were maintained on mitotically inactivated mouse embryonic fibroblast (MEF) feeder cells with culture medium containing Knockout DMEM (Life Technologies, catalog number: 10829-018) supplemented with 10% (vol/vol) Knockout Serum Replacement (KSR; Life Technologies, catalog number: 10828-028), 10% (vol/vol) human plasmanate (Talecris Biotherapeutics), 1% (vol/vol) non-essential amino acids (NEAA), 1% (vol/vol) Gluta-MAX, 1% (vol/vol) penicillin streptomycin, and 55 μM 2-mercaptoethanol (Chang et al., 2013). The medium was supplemented with basic fibroblast growth factor (bFGF; 30 ng/mL) and exchanged with fresh medium daily. Cells were enzymatically detached using Accutase (Millipore) and regularly passaged upon reaching approximately 80% confluence.

Prior to cell seeding, both mineralized and non-mineralized matrices were coated with Matrigel (Corning, catalog number: 354277) diluted with DMEM (Invitrogen) at a ratio of 1:82 (298 μL Matrigel diluted with 24.5 mL DMEM) and incubated overnight at 4°C. The following day, matrices were incubated in medium containing high glucose DMEM, 20% (vol/vol) fetal bovine serum [Premium (FBS); Atlanta Biologicals, catalog number: S11150], and 1% (vol/vol) penicillin streptomycin for 24 h at 37°C. Cells were seeded at an initial density of 10,000 cells/cm^2^ and cultured in hESC-maintenance medium containing bFGF (30 ng/mL) for 1 day. The hESCs were cultured for an additional 2 days in hESC-maintenance medium without bFGF. The hESCs were subsequently cultured in growth medium (GM) containing high glucose DMEM, 4 mM l-glutamine, 10% (vol/vol) FBS (Gibco), and 1% (vol/vol) penicillin streptomycin. Adenosine and PSB 603 were supplemented into growth media for cell treatment. Adenosine (Sigma Aldrich) was first dissolved in DMEM as 11.2 mM stock solution and filter sterilized by using 0.22-μm syringe filters. The stock solution was further diluted 1,000× in growth media to 11.2 μM as the final concentration for experiments. 8-[4-[4-(4-chlorophenzyl)piperazide-1-sulfonyl)phenyl]]-1-propylxanthine (PSB 603) (Tocris Biosciences, catalog number: 3198) was dissolved in dimethyl sulfoxide (DMSO) as 1 mM stock solution and filter sterilized. The stock solution was further diluted 10,000× in growth media to 100 nM as the final concentration for experiments. All cell cultures were maintained at 37°C and 5% CO_2_.

### Cell Tracker Staining

To visualize cell attachment in 2-D culture, cells were stained with CellTracker (Life Technologies, catalog number: C34552) at 3, 5, and 8 days postseeding. Cells attached to the matrix were stained in 20 μM CellTracker reagent in DMEM at 37°C for 30 min. The stained cells were imaged using a fluorescence microscope (Carl Zeiss, Axio Observer.A1).

### Quantitative Real-Time Polymerase Chain Reaction

Samples from two biological experiments were collected and pooled together using TRIzol Reagent (Life Technologies, catalog number: 15596-018), and RNA extraction was performed using phenol–chloroform extraction method. For each sample, 1 μg of RNA was reverse transcribed to complementary DNA (cDNA) using iScript cDNA Synthesis Kit (Bio-Rad, catalog number: 17-8891) according to the manufacturer’s instructions. The synthesized cDNA was analyzed via quantitative real-time polymerase chain reaction (qRT-PCR) for osteogenic markers, such as OCN, runt-related transcription factor 2 (RUNX2), and secreted phosphoprotein 1 (SPP1) as well as additional genes, including solute carrier family 20 (phosphate transporter), member 1 (SLC20a1), and Nanog homeobox (NANOG). Primer sequences for each analyzed gene are provided in Table S1 in Supplementary Material. Reactions were performed using SYBR Select Master Mix (Life Technologies, catalog number: 4472908) and ABI Prism 7700 Sequence Detection (Applied Biosystems). Fold expression values were determined by $2^{-\Delta \Delta C_{\text{t}}}$ after normalizing each target gene with respect to the housekeeping gene (GAPDH) within the sample, and compared to undifferentiated hESCs as the control that was expressed as 1.

### Immunofluorescent Staining

Osteogenic differentiation of hESCs was evaluated by immunofluorescent staining for OCN. Cells were fixed in 4% paraformaldehyde for 10 min at RT, and incubated in blocking buffer composed of 3% (wt/vol) bovine serum albumin (BSA) and 0.1% (vol/vol) Triton™ X-100 in PBS for 45 min. The fixed cells were incubated overnight with primary antibodies (1:50; mouse monoclonal, Santa Cruz Biotechnology, catalog number: sc-74495) in blocking buffer at 4°C. They were then incubated with blocking buffer containing secondary antibody (1:100; goat anti-mouse Alexa Fluor^®^ 568, Life Technologies, catalog number: A-11004) and phalloidin (1:100; Alexa Fluor^®^ 488, Life Technologies, catalog number: A12379). Nuclei were counter-stained using Hoechst 33342 (2 μg/mL; Life Technologies, catalog number: H1399) at RT for 10 min and washed with PBS. The samples were mounted onto glass slides and imaged using a fluorescence microscope. Images were acquired using an A1 Zeiss Inverted microscope and analyzed using ImageJ. Immunofluorescent images of all samples were acquired under the linear mode and at an exposure time of 1 s. The background was uniformly subtracted from all images using a rolling ball radius method and value of 750.0 pixels.

### Statistical Analysis

Statistical analyses were carried out using GraphPad Prism^®^ (v. 5.00). One-way analysis of variance (ANOVA) along with Tukey–Kramer *post hoc* test was used to compare multiple groups at the same time point. Two-tailed Student’s *t*-test was utilized to compare two groups at the same time point. Two-way ANOVA with Bonferroni *post hoc* test was used to compare multiple groups at different time points. From these tests, the *p*-values were determined and asterisks were assigned to denote statistical significances for *p*-values <0.05.

## Results

### Synthesis and Characterization of Non-Mineralized and Mineralized PEGDA-*co*-A6ACA Matrices

**Figure 1:**
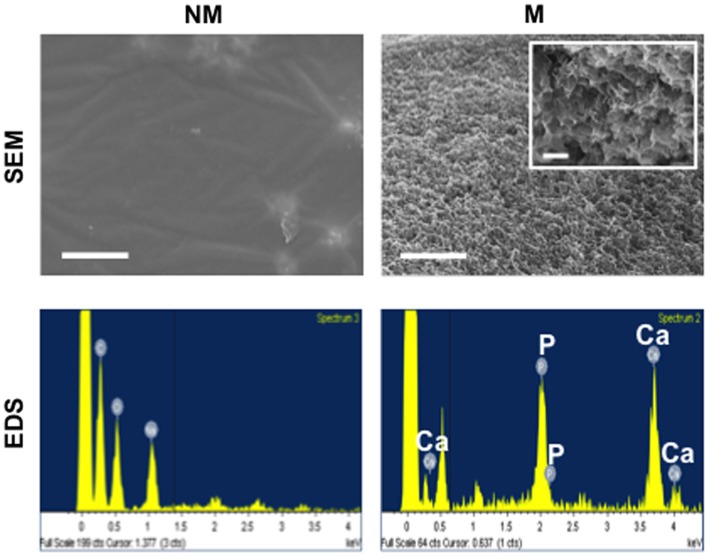
**Characterization of non-mineralized and mineralized matrices**. Scanning electron microscopy (SEM) images and corresponding energy dispersive spectroscopy (EDS) of non-mineralized (NM) and mineralized (M) matrices. Scale bars represent 5 μm. Inset shows high magnification SEM image. Scale bar represents 500 nm.

Hydrogel matrices were synthesized by cross-linking poly(ethylene glycol)-diacrylate (PEGDA) with *N*-acryloyl 6-aminocaproic acid (A6ACA). Mineralization of matrices occurred with the binding of Ca^2+^ to terminal carboxyl groups along the pendant side chain of A6ACA, leading to the subsequent nucleation and growth of CaP minerals (Phadke et al., 2010). In order to characterize the morphology and elemental composition mineralized matrices, SEM and energy dispersive spectra (EDS) analyses were performed as shown in Figure 1. The SEM images showed the mineralized matrices displaying a continuous layer of bound CaP minerals exhibiting a plate-like morphology. The elemental spectra analysis confirmed the presence of calcium and phosphorous elements in the mineralized matrices with a quantified Ca/P ratio of approximately 1:31. As expected, no such moieties or peaks distinguishing such elements were observed for non-mineralized matrices.

### Mineralized Matrix-Driven Osteogenic Differentiation of hESCs

**Figure 2:**
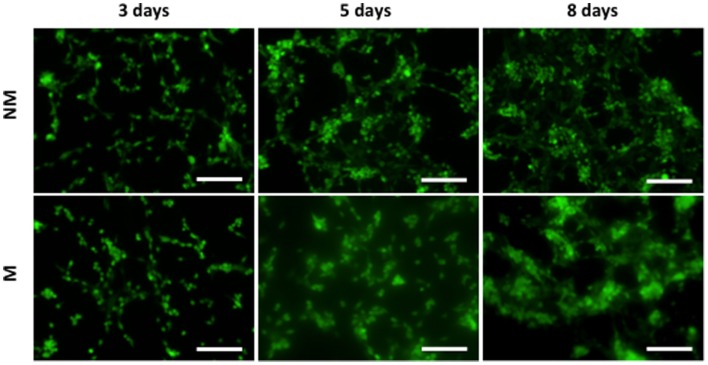
**Morphology of hESCs on non-mineralized and mineralized matrices**. Fluorescent images of hESCs stained by CellTracker cultured on non-mineralized (NM) and mineralized (M) matrices after 3, 5, and 8 days of culture. Scale bars represent 200 μm.

**Figure 3:**
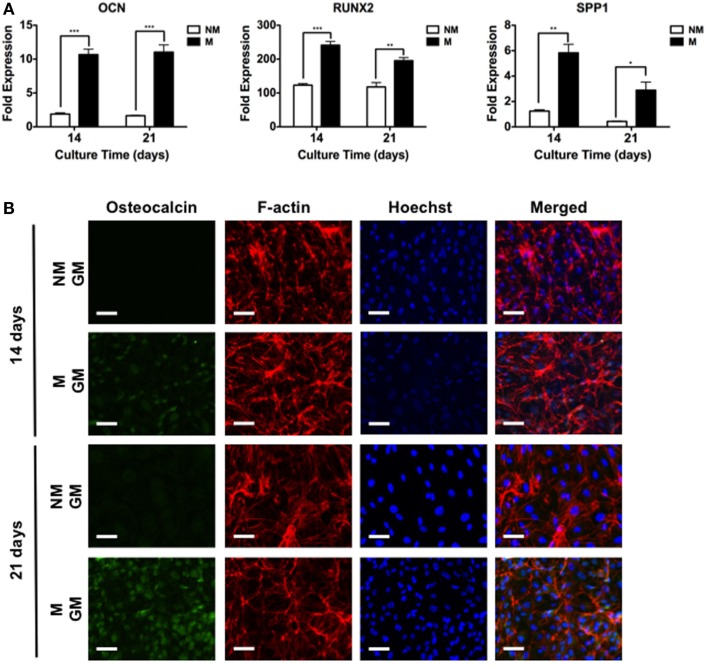
**Mineralized matrix-mediated osteogenic differentiation of hESCs**. **(A)** Gene expression profiles for OCN, RUNX2, and SPP1 of hESCs on non-mineralized (NM) and mineralized (M) matrices after 14 and 21 days of culture. **(B)** Immunofluorescent staining of osteocalcin, F-actin, and Hoechst for hESCs cultured on NM and M matrices in growth medium (GM) after 14 and 21 days. Data are presented as mean ± SEs (*n* = 3). Two-tailed Student’s *t*-test was used to compare two groups at the same time point. Asterisks denote statistical significances according to *p*-values (*p < 0.05, **p < 0.01, ***p < 0.001). Scale bars represent 100 μm.

The hESCs cultured on non-mineralized and mineralized matrices in GM were able to adhere and grow on the matrices as a function of time (Figure 2). Analysis of the gene expression demonstrated a significant upregulation of various osteogenic markers, OCN, RUNX2, and SPP1 for hESCs cultured on mineralized matrices compared to non-mineralized matrices over 21 days (Figure 3A). This is consistent with our previous study, which showed significant upregulation of osteogenic markers in hESCs cultured on mineralized matrices (Kang et al., 2014b). Immunofluorescent staining for OCN further corroborated these findings, where OCN was stained positive in hESCs cultured on mineralized matrices in contrast to those cultured on non-mineralized matrices (Figure 3B). OCN staining intensity markedly increased between 14 and 21 days. In addition to osteogenic expression, hESCs exhibited a higher upregulation of the sodium–phosphate symporter, SLC20a1, when cultured on mineralized matrices compared to non-mineralized matrices (Figure S1A in Supplementary Material). Consistent with the differentiation of hESCs, the pluripotency marker, NANOG, was found to be downregulated as a function of time for both non-mineralized and mineralized matrix groups (Figure S1B in Supplementary Material). The hESCs cultured on mineralized matrices showed higher upregulation of A2bR compared to non-mineralized matrices (Figure S2 in Supplementary Material).

### Mineralized Matrix-Assisted Osteogenic Differentiation Through A2bR Signaling

**Figure 4:**
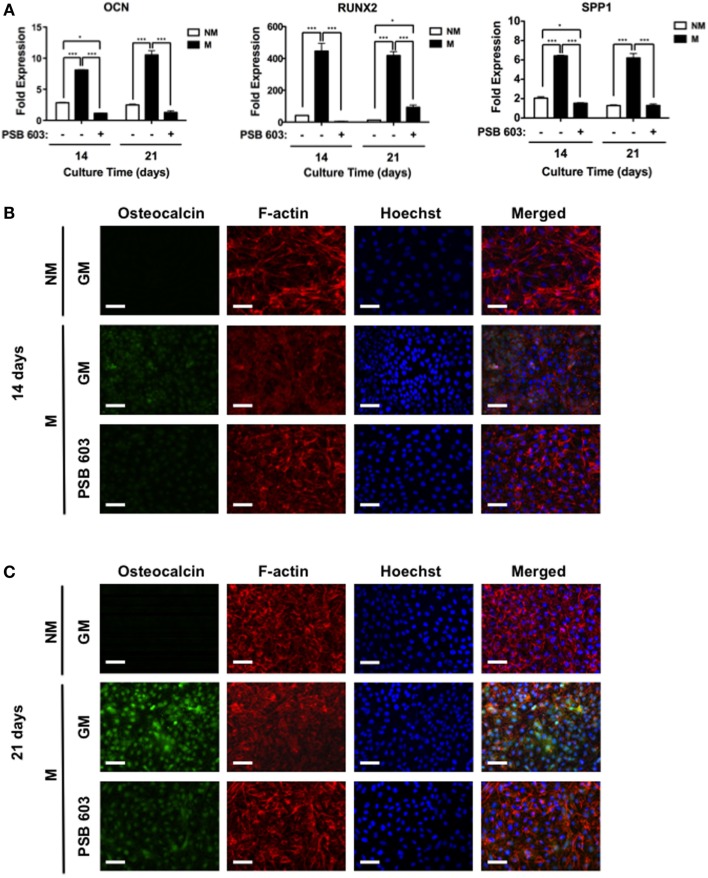
**Mineralized matrix-assisted osteogenic differentiation of hESCs involves A2bR**. **(A)** OCN, RUNX2, and SPP1 gene expressions of hESCs on non-mineralized (NM) in growth medium (GM) as well as on mineralized matrices in growth medium (GM) with and without A2bR antagonist, PSB 603. Corresponding immunofluorescent staining of osteocalcin, F-actin, and Hoechst for hESCs cultured on NM and M matrices as well on M matrices in presence of PSB 603 for **(B)** 14 and **(C)** 21 days of culture. Data are presented as mean ± SEs (*n* = 3). Multiple groups at the same time point were compared by one-way ANOVA with Tukey–Kramer *post hoc* test. Asterisks denote statistical significances according to *p*-values (*p < 0.05, ***p < 0.001). Scale bars represent 100 μm.

To explore whether adenosine signaling through A2bR is involved during osteogenic differentiation of hESCs on mineralized matrices, we used a selective antagonist, 8-[4-[4-(4-chlorophenzyl)piperazide-1-sulfonyl)phenyl]]-1-propylxanthine (PSB 603), to block A2bR. As shown in Figure 4A, the presence of PSB 603 in the culture medium abrogated the mineralized matrix-induced upregulation of osteogenic genes (Figure 4A). These observations were further supported by analysis of immunofluorescent staining intensity for OCN. The intensity of OCN was increased for hESCs cultured on mineralized matrices after 14 days, but diminished in the presence of PSB 603 (Figure 4B). A similar finding was observed at 21 days post-culture (Figure 4C).

### Exogenous Adenosine Promotes Osteogenic Differentiation Through A2bR

**Figure 5:**
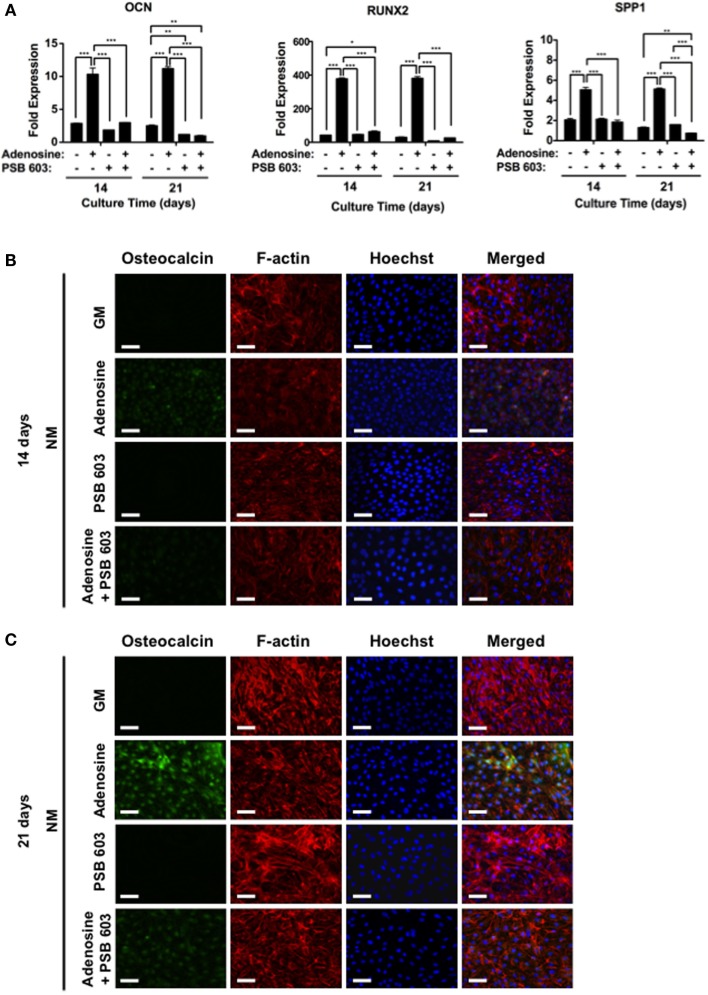
**Exogenous adenosine-mediated osteogenic differentiation of hESCs through A2bR signaling**. **(A)** OCN, RUNX2, and SPP1 gene expressions for hESCs on non-mineralized (NM) matrices in growth medium (GM) supplemented with or without adenosine and PSB 603. Corresponding immunofluorescent staining of osteocalcin, F-actin, and Hoechst for hESCs on NM matrices after **(B)** 14 and **(C)** 21 days of culture. Data are presented as mean ± SEs (*n* = 3). Multiple groups at the same time point were compared by one-way ANOVA with Tukey–Kramer *post hoc* test. Asterisks denote statistical significances according to *p*-values (*p < 0.05, **p < 0.01, ***p < 0.001). Scale bars represent 100 μm.

To further substantiate the role of adenosine signaling on osteogenic differentiation of hESCs, cells were cultured on non-mineralized matrices, devoid of CaP, and exposed to culture medium containing adenosine. Results showed supplementation of exogenous adenosine-promoted osteogenic differentiation of hESCs on non-mineralized matrices, while inhibition of A2bR with PSB 603 led to a downregulation of the osteogenic genes, OCN, RUNX2, and SPP1 (Figure 5A). In addition, immunofluorescent staining for OCN was highly positive for cells cultured on non-mineralized matrices in the presence of adenosine, whereas a reduction in the staining intensity was observed in other medium conditions after 14 and 21 days (Figures 5B,C).

## Discussion

Previously, we have shown that human PSCs, including hESCs, can be differentiated into osteoblasts by using matrix-based cues from the mineralized biomaterials (Kang et al., 2014a,b). Consistent with these findings, the hESCs on mineralized matrices exhibited an upregulation of osteogenic markers in GM even in the absence of any osteogenic-inducing soluble factors. As evident from the NANOG expression, the cells on both mineralized and non-mineralized matrices lost their pluripotency, while only these on mineralized matrices underwent significant osteogenic differentiation. The observed loss of pluripotency on all matrices is due to the culture conditions, which lack components that are known to assist maintenance of pluripotency of hESCs (Chang et al., 2013). Since increased surface roughness at the cell–material interface may increase osteogenic differentiation (Faia-Torres et al., 2014), a limitation to this study is the extent of how the topology of mineralized matrices contributes to osteogenic commitment.

The hESCs on mineralized matrices not only showed an upregulation of gene markers that are relevant to osteogenic differentiation but also exhibited high levels of OCN, a bone-specific protein. The cells on non-mineralized matrices, which lack any intrinsic ability to induce osteogenesis, underwent osteogenic differentiation when cultured in medium supplemented with adenosine. Both mineralized matrix- and exogenous adenosine-assisted osteogenic differentiations of hESCs were annulled in the presence in PSB 603, which is a known pharmacological inhibitor of A2bR. Taken together, these results underscore the influence of adenosine signaling through A2b receptor on osteogenic differentiation of hESCs. These findings are also consistent with our previous reports that showed the role of adenosine signaling on mineralized matrix-induced osteogenic differentiation of hMSCs (Shih et al., 2014). The adenosine signaling mediated by the mineralized matrices not only promotes osteogenic differentiation of hMSCs but also inhibits their adipogenic differentiation (Kang et al., 2015).

The observation that hESCs on the mineralized matrix exhibited upregulation of SLC20a1 implies the potential contribution of ${\text{PO}}_{4}^{3-}$ ions from the CaP minerals toward this process. SLC20a1, also known as PiT-1, is a sodium–phosphate symporter that transports ${\text{PO}}_{4}^{3-}$ ions from the extracellular milieu into the cytoplasm. We have previously shown that the extracellular ${\text{PO}}_{4}^{3-}$ of the CaP minerals play an important role in promoting osteogenic differentiation of hMSCs through adenosine signaling, where the cellular intake of ${\text{PO}}_{4}^{3-}$ is regulated by SLC20a1 (Shih et al., 2014). Previous studies have also shown the importance of SLC20a1 in mineralization (Yoshiko et al., 2007; Cowan et al., 2012) as well as A2bR in osteogenic differentiation (Gharibi et al., 2011; He et al., 2013). Despite the differences in cell machinery, we find hESCs consistently differentiate into osteoblasts and upregulate the phosphate transporter, SLC20a1, on CaP matrices in a similar manner to hMSCs. Such an upregulation may facilitate ${\text{PO}}_{4}^{3-}$ to serve as a substrate for the production of adenosine triphosphate (ATP), which has been shown to be essential for osteogenic differentiation (Chen et al., 2008). This production of ATP results in a subsequent increase in extracellular adenosine that requires A2bR signaling during CaP-directed osteogenic differentiation (Shih et al., 2014). Although we did not investigate its role, calcium is required during ${\text{PO}}_{4}^{3-}$-induced osteogenic differentiation since this process is blunted in the absence of CaP crystal formation (Khoshniat et al., 2011).

We detected A2bR gene expression in pluripotent hESCs that increased as a function of culture time, possibly demonstrating the increased role of the receptor during the maturation process. Despite the finding that mineralized matrices promote osteogenic differentiation of hESCs through A2bR expression, the downstream signaling following A2bR expression remains unclear. For instance, whether A2bR signaling crosstalks with calcium-responsive ion channels in ESCs remains to be determined. Studies have shown A2bR expression resulted in significant potentiation of P-type Ca^2+^ current (Vacas et al., 2003). A2bR signaling is coupled to the activation of cyclic AMP (cAMP) through *G*_s_ proteins, leading to stimulation of downstream signaling molecules (Ham and Evans, 2012). Furthermore, Hsiao et al. (2008) have demonstrated the engineering of a G-protein coupled receptor (GPCR) with constitutive *G*_s_ signaling in murine osteoblasts dramatically enhanced bone mass. A study by Siddappa et al. (2008) reported the involvement of protein kinase A (PKA), where cAMP/PKA signaling induced *in vitro* osteogenesis of hMSCs and promoted *in vivo* bone formation with cAMP either stimulating or inhibiting osteogenesis in hMSCs, depending on the duration rather than the strength of the signal (Siddappa et al., 2010). However, cAMP may exert contrasting effects during early versus late stages of osteogenic differentiation of murine ESCs by inhibiting differentiation at early stages, but is required at later stages (Zhang et al., 2012).

To summarize, the results in this study demonstrate that CaP-bearing biomineralized PEGDA-*co*-A6ACA matrices direct osteogenic commitment of hESCs via adenosine signaling. Although this study describes the importance of adenosine signaling through A2bR in directing ESC osteogenic fate, there is warrant for future studies to further elucidate the role of calcium and the signaling network that are involved in mediating osteogenic differentiation of PSCs on CaP matrices. Further efforts to investigate the cellular signaling pathways can shed light on our biological understanding of how cells behave in biomaterials to expose new targets of regulation and aid in the development of novel biomaterials for cell transplantation and bone tissue engineering.

## Author Contributions

VR, YS, HKang, HKabra, and SV designed the experiments. VR, YS, HKang, and HKabra, performed the experiments. VR, YS, HKang, HKabra, and SV analyzed and interpreted the data. VR, YS, and SV wrote the paper.

## Conflict of Interest Statement

The authors declare that the research was conducted in the absence of any commercial or financial relationships that could be construed as a potential conflict of interest.
