# Supplementary material for: Adenosine Signaling Mediates Osteogenic Differentiation of Human Embryonic Stem Cells on Mineralized Matrices
Source: Front Bioeng Biotechnol. 2015 Nov 10;3:185. doi: 10.3389/fbioe.2015.00185 (PMC4639610; doi:10.3389/fbioe.2015.00185)
Supplement: Supplementary file 1 [file Data_Sheet_1.PDF]

Supplementary Material

Adenosine signaling mediates osteogenic differentiation of human embryonic stem cells on mineralized matrices

Vikram Rao[1#], Yu-Ru V. Shih[1#], Heemin Kang[2], Harsha Kabra[1], and Shyni Varghese[1*]

[1] *Department of Bioengineering, University of California, San Diego, La Jolla, CA 92093, USA.*
[2] *Materials Science and Engineering Program, University of California, San Diego, La Jolla, CA 92093, USA.*

* **Correspondence**: Department of Bioengineering, University of California, San Diego, 9500 Gilman Drive, Mail Code 0412, La Jolla, CA 92093-0412, USA. Tel.: +1 858 822 7920; Fax: +1 858 534 5722. *E-mail: svarghese@ucsd.edu (S. Varghese)*

# indicates both authors contributed equally to this work.

Supplementary Table 1. Sequences of forward and reverse primers used in qRT-PCR experiments.

Gene	Primer Sequence
Glyceraldehyde 3-phosphate dehydrogenase (GAPDH)	Forward: 5´ CAT CAA GAA GGT GGT GAA GC 3´
	Reverse: 5´ GTT GTC ATA CCA GGA AAT GAG C 3´
Osteocalcin (OCN)	Forward: 5´ GAA GCC CAG CGG TGC A 3´
	Reverse: 5´ CAC TAC CTC GCT GCC CTC C 3´
Runt-related transcription factor 2 (RUNX2)	Forward: 5´ CCA CCC GGC CGA ACT GGT CC 3´
	Reverse: 5´ CCT CGT CCG CTC CGG CCC ACA 3´
Secreted phosphoprotein 1 (SPP1)	Forward: 5´ AAT TGC AGT GAT TTG CTT TTG C 3´
	Reverse: 5´ CAG AAC TTC CAG AAT CAG CCT GTT 3´
Solute carrier family 20 (phosphate transporter), member 1 (SLC20a1)	Forward: 5´ TCC CAT CAG TAC AAC ACA TTG TAA A 3´
	Reverse: 5´ CAG TCA ACA GCC TTC TTG GA 3´
Nanog homeobox (NANOG)	Forward: 5´ GAT TTG TGG GCC TGA AGA AA 3´
	Reverse: 5´ ATG GAG GAG GGA AGA GGA GA 3´
Adenosine 2b receptor (A2bR)	Forward: 5´ TCT GTG TCC CGC TCA GGT AT 3´
	Reverse: 5´ GTC AAT CCG ATG CCA AAG GC 3´

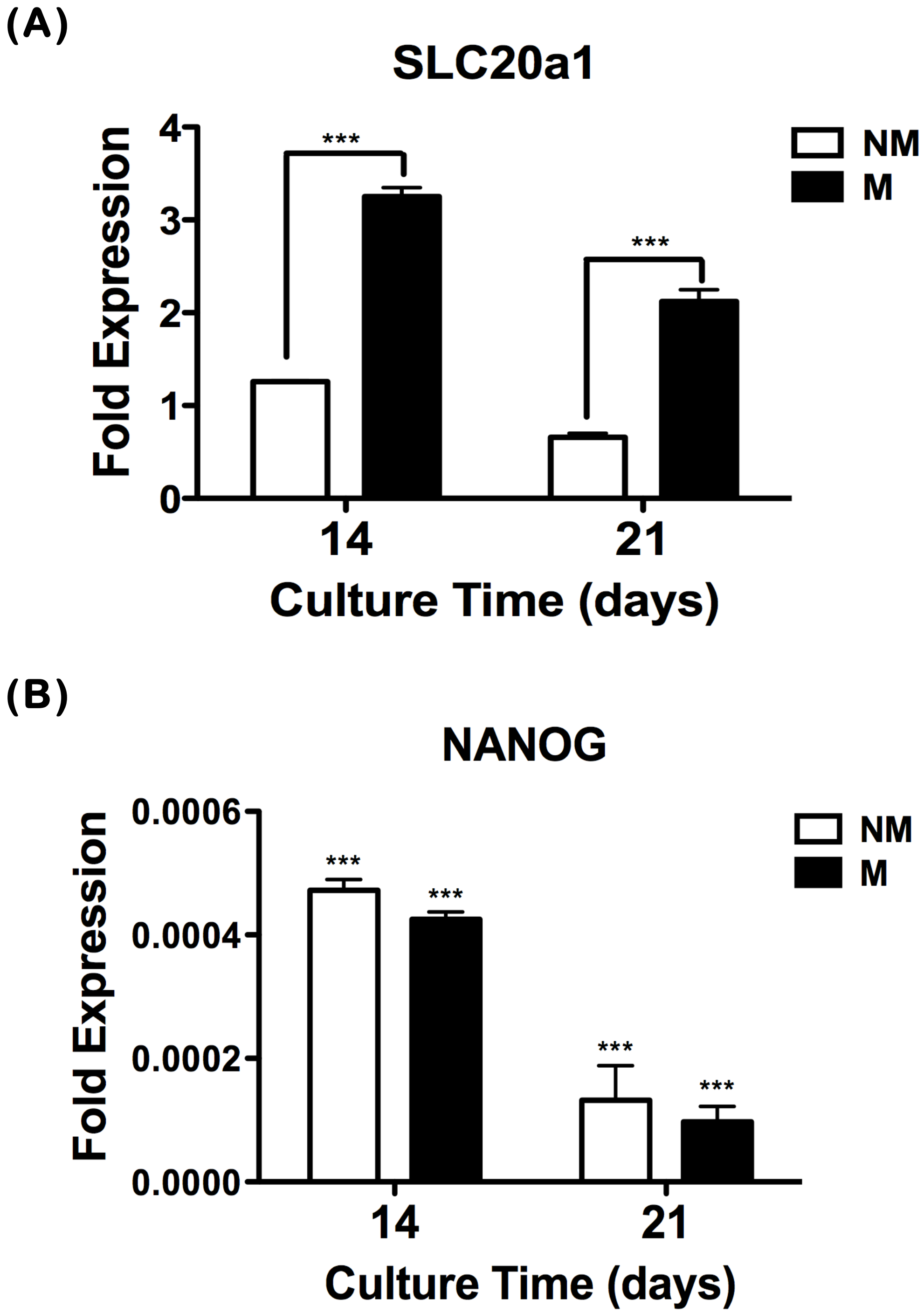

Supplementary Figure 1. (A) Solute carrier family 20 (phosphate transporter), member 1 (SLC20a1) gene expression for hESCs cultured on non-mineralized (NM) and mineralized (M) matrices. Two-tailed Student's *t*-test was used to compare two groups at the same time point. (B) NANOG gene expression for hESCs cultured on NM and M matrices. Two-way ANOVA with Bonferroni *post-hoc* test was used to compare multiple groups at different time points to the undifferentiated hESCs. Data are presented as mean ± standard errors ($n = 3$). Asterisks denote statistical significances according to *p*-values (***: $p < 0.001$).

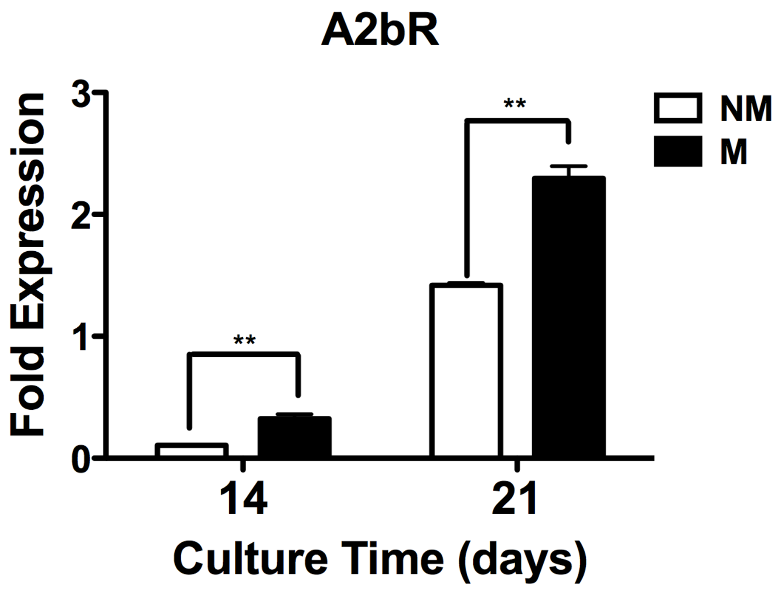

Supplementary Figure 2. Adenosine 2b receptor (A2bR) gene expression for hESCs cultured on non-mineralized (NM) and mineralized (M) matrices. Two-tailed Student's *t*-test was used to compare two groups at the same time point. Data are presented as mean ± standard errors ($n = 3$). Asterisks denote statistical significances according to *p*-values (**: $p < 0.01$).
